# Mondino de Luzzi: a luminous figure in the darkness of the Middle Ages

**DOI:** 10.3325/cmj.2014.55.50

**Published:** 2014-02

**Authors:** Alexandra Mavrodi, George Paraskevas

No research on the history of anatomy in the medieval period can be considered complete without mentioning Mondino de Luzzi (cca. 1270-1326). Mondino de Luzzi (also known as Mundini or Mundinus, Liuzzi, Lucci, Liucius, or even Lentiis and Leutiis) was an Italian physician, anatomist, and professor of surgery at the University of Bologna (Figure 1) (1). While his admirers call him the “Restorer of anatomy,” and his teachings on dissection influenced even Leonardo da Vinci (2), his critics claim that he only observed rather than performed dissections and purely repeated his predecessors’ findings (3). All this controversy has justifiably created an atmosphere of mystery around this medieval anatomist. Therefore, it is necessary to clarify the life and achievements of Mondino de Luzzi.

**Figure 1:**
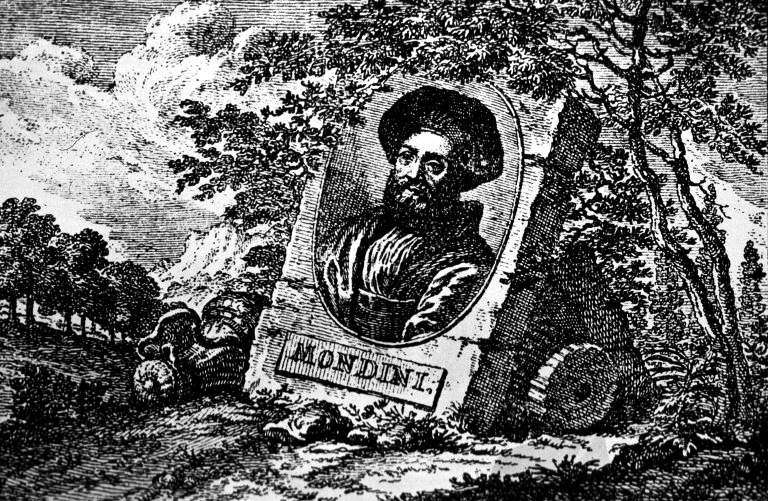
Portrait of Mondino created by Giovanni Alessandro Brambilla (Available from: *http://ihm.nlm.nih.gov/luna/servlet/detail/NLMNLM~1~1~101436495~174950:Mondino-De-Luzzi*).

## Historical context

Since in the early Middle Ages, between 9th and 11th century, the development of rational thought and investigation was paralyzed by the Church authorities, physicians could only repeat the doctrines of the major figures of the past, such as Aristotle or Galen, without questioning them. Anatomical dissection was illegal, so the Galenic work constituted the most complete description of the human body (4).

**Figure 2:**
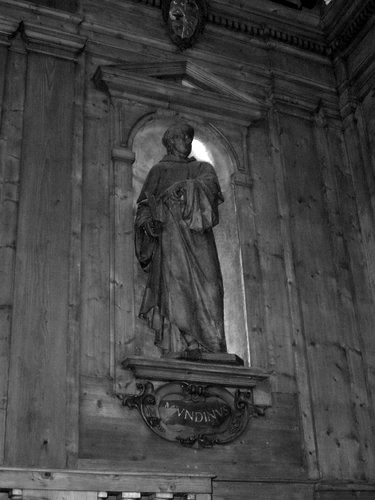
Mondino’s statue among other statues of major personalities from the history of medicine created by Silvestro Giannotti in the anatomical theater of the Palazzo dell' Archiginnasio at the University of Bologna (the photograph published with the permission of Lucca Borghi from Himetop – The History of Medicine Topographical Database).

The first progress was made only in the 12th century when several universities were established, such as those in Padua, Montpellier, Oxford, and Bologna (5), where Mondino obtained his medical degree and spent his teaching career. The University of Bologna was extremely popular, attracting students from the whole Italy and many other countries (5) (Figure 2). Consequently, in 1292 it was granted a bull by Pope Nicolas II, which permitted all doctors having graduated from Bologna to teach in any University in the world (6).

The progress of medicine, and especially anatomy, was accelerated by the rule of Frederick II (1194-1250), Emperor of the Holy Roman Empire. In 1231, Frederick II decreed that medical schools were allowed to dissect at least one human body each five years (7). However, until relatively recently, historians of medicine believed that in the Middle Ages human dissection had not been permitted, probably due to a misrepresentation of the bull issued by Pope Boniface VIII around 1300, which declared that anybody “cutting up bodies of the dead and boiling them so as to separate the bones” would be excommunicated. At first glance, it does seem that this referred to human dissection, but after carefully reading the entire bull and taking into account historical events of the time, it is clear that it actually banned dismembering and boiling bodies of the dead during the crusades to preserve them for burial at home (6).

## Mondino de Luzzi and the first recorded dissection

About the same time of Frederick’s decree and Pope’s Boniface bull, Mondino de Luzzi was preparing for the career of a physician. Born in Bologna about 1270, he grew in a rich bourgeois family. His father, Nerino Fronzoli, owned a pharmacy, where Mondino worked (8). His uncle, Liuccio di Luzzi, was a professor of physics, philosophy and medicine at the University of Bologna (7). If Mondino chose medicine because of his uncle, he chose anatomy because of his teacher, Taddeo di Alderotto (9). Mondino got one step ahead of his predecessor and began to practice dissections as a part of the course of anatomy. This made him known as the first person to perform a public human dissection after Herophilus and Erasistratus. The dissection took place in 1315 on an executed criminal, most likely a woman and was observed by medical students and the public with the purpose to indicate the exact position of the anatomical elements described by Galen (10). Although this was a historical landmark, it seems that human dissection had already been performed before, since it is undeniable that during this whole period surgeons performed post-mortem examinations with the purpose of ascertaining the cause of death (2,11).

Mondino lead the dissection from a chair situated on a podium (12) and read aloud from Galen’s books (2). If the findings did not match the descriptions, they were interpreted as morphological transmutation (13). For this reason, Mondino has been repeatedly accused of not recognizing the anatomical errors made by Galen. The practical part of the dissection was performed by his assistants. The demonstrator dissected the cadaver and the ostensor used a wand to indicate the position of the anatomical elements (10). It seems that the demonstrator actually witnessed more anatomy than the professor himself (14). This is why some historians doubt whether Mondino ever actually dissected himself. The answer to this question is of major importance because it defines the value of Mondino’s contribution to the history of human anatomy. There are paintings of Mondino *ex cathedra*, but they were created many years after Mondino’s death. What we know for sure is that Mondino dissected at least two human female cadavers (12). In his book “Anathomia” he clearly stated that he “anatomized” (“anatomizavi”) (11), as well as “videre ad sensum” (“to see according to practice”) (15). The book clearly and elaborately explains the dissecting procedure, as well as techniques used to visualize specific anatomical structures and tools used to dissect. Except for using blades and knives, Mondino stated that he boiled parts of the corpse to separate the bones or that he dissected the body after putting it into water to achieve better visualization of muscles and nerves (16). Besides this, Guy de Chauliac, one of his pupils and “father of modern French surgery,” claimed that Mondino dissected multiple cadavers (17,18). Taking everything into consideration, a likely scenario is that Mondino dissected himself more often initially and later only occasionally. This theory explains the presence of his assistants in the mature stage of his career.

In Mondino’s era nothing concerning the process of dissection was easy. One of the most difficult parts was to find a cadaver. For his dissections Mondino de Luzzi used cadavers of criminals (9). Although the local public authorities provided some cadavers to the medical school of Bologna, there must have also been unofficial dissections (19). For instance, in 1319 four students were arrested for stealing a corpse from the grave and bringing it to their Master Alberto, a lecturer at the University of Bologna (20). When a cadaver was obtained, there ensued a fight against time, because there were no means of preservation. This is why abdominal cavity, which contained organs that putrefied most easily, was dissected first, followed by the thorax, head, and extremities. In fact, one day was dedicated to each of these regions and as a result the dissection lasted four days (9), even including the nights. Another means to prevent rapid putrefaction was to perform dissections in the coldest days of the year. For this reason, dissections were scheduled in January or February and were combined with the Carnival (13), when the school would provide food and wine for the students in order to create a more acceptable atmosphere (2).

## “Anathomia”

Mondino’s book “Anathomia” was finished round 1316 (21). For at least two centuries, it remained a classical anatomical textbook used by all European universities (22). The book is a treatise on human anatomy and constitutes a practical manual of dissection, including also some physiological information (16,23,24), One of this book’s innovations was the specification of the basic elements of organ anatomy: the position in a topographic region of the body, relationship with the surrounding structures, shape, size, texture, parts, physiology, and pathology (16). Names of various anatomical features were in Latin accompanied with Arabic (25). The structure of the book follows the order of dissection, starting from the abdominal cavity and ending with the head (9).

Due to Mondino’s adhesion to the doctrines of the past, “Anathomia” contained three types of errors: the errors that had initially been made by Galen and repeated by Mondino, the errors made by Mondino due to misinterpretation of Galen’s works, and the errors made by Mondino in an effort to effectively combine the Aristotle’s and Galen’s observations (19). However, Mondino did not just repeat the claims of his predecessors, but formulated a few theories of his own. Such was the theory on Galen’s concept of a complicated network of fine arteries below the base of the brain, which Mondino named “*rete mirabile*” or “marvelous network.” Mondino disagreed with Galen about its function, believing that its altered operation was related to sleep (26). Additionally, Mondino must have discovered the principal excretory duct of the pancreas, later named after Wirsung, without identifying its accurate route and function (27). Moreover he was the first to use the term “mesenterium” and explained the terms describing the parts of the small intestine (13).

“Anathomia” significantly contributed to the development of neuroanatomy, although the chapter on the anatomy of the head is relatively short, probably because it was considered a sin to open the skull (25). Mondino described the dura and the pia matter from today’s three cerebral membranes and associated the choroid plexus with the ability of thinking (16). He also attributed many of the functions of the brain to the cerebral ventricles, slightly detaching from Galen, who gave more importance to the brain parenchyma. After having divided the lateral ventricles in three parts, he associated the anterior part with fantasy, the middle with special senses, and the posterior with imagination. He also attributed the power of cognition and prognostication to the third ventricle and the function of memory to the fourth (28).

Regarding the heart, Mondino detached from Galen’s notion that it consisted of two chambers, in line with Aristotle’s view of a three chambered heart. In his work, the function of the presumable middle ventricle, which consisted of many cavities, was associated with the conversion of blood into the “vital spirit” (16).

Although Galen believed that the uterus consisted of two cavities, according to Mondino there were seven of them. This was probably the influence of Byzantine medicine, which attributed mystic qualities to the number seven. Three warmer cavities were intended for male fetuses, three colder ones for female fetuses, and the seventh was intended for a hermaphrodite (19). This is peculiar because Mondino dissected at least two female corpses and described the shape, position, changeable size, and the inner morphology of the uterus with great accuracy (16).

## Conclusion

In spite of the views of his critics, we can conclude that Mondino de Luzzi made important and innovative contributions to the medieval anatomical science. He was not a blind follower of Galen, since he detached from his predecessor in many topics. Even though Mondino was not the first to perform a dissection, his work marked the beginning of a new era, when dissection was incorporated in the curriculum of medical schools. Keeping all this in mind, Mondino fairly deserves the title of the “Restorer of Anatomy,” who paved the way for the great discoveries of the future. As Dr Ernest Wickersheimer, a prominent French historian of medicine, claims: “if there was an actual Renaissance of anatomy in Western Europe, it was due to Mondino de Luzzi, who signaled the beginning of a new era in the study of the human body” (29).
